# Towards optimizing the host response to biotics: a report of an International Scientific Association for Probiotics and Prebiotics working group

**DOI:** 10.1080/19490976.2026.2728828

**Published:** 2026-09-08

**Authors:** Geoffrey A. Preidis, Premysl Bercik, Andre Marette, Liam O'Mahony, Vanessa Sperandio, Eamonn MM Quigley

**Affiliations:** a Division of Gastroenterology, Hepatology & Nutrition, Department of Pediatrics, Baylor College of Medicine and Texas Children's Hospital, Houston, TX, USA; b USDA/ARS Children's Nutrition Research Center, Baylor College of Medicine, Houston, TX, USA; c Farncombe Family Digestive Health Research Institute, McMaster University, Hamilton, ON, Canada; d Quebec Heart and Lung Institute (IUCPQ), Laval University, Québec, QC, Canada; e Institute of Nutrition and Functional Foods (INAF), Laval University, Québec, QC, Canada; f Department of Medicine, School of Microbiology, APC Microbiome Ireland, University College Cork, Cork, Ireland; g Department of Medical Microbiology and Immunology, University of Wisconsin–Madison, Madison, WI, USA; h Lynda K. and David M. Underwood Center for Digestive Health, and Houston Methodist and Weill Cornell Medical College, Houston, TX, USA

**Keywords:** Prebiotic, probiotic, microbiome, metabolome, immune response, host metabolism, gut–brain axis, enteric nervous system

## Abstract

This report summarizes the deliberations of a working group convened by the International Scientific Association for Probiotics and Prebiotics (ISAPP) at their 2025 annual meeting to identify the key targets that mediate the host response to biotics. Probiotics, prebiotics, synbiotics, and postbiotics have been explored in numerous contexts, but results of clinical trials are mixed. In most studies performed to date, a therapeutic product and its dose and administration strategy were selected based on factors other than rigorous mechanistic evidence. Given recent advances in our understanding of interactions between biotics, the host, and the gut microbiome, we sought to determine whether a more rational, informed approach might now guide the more precise selection of a biotic for a specific indication. The panel addressed biotic modulation of the immune system, host metabolism, the enteric nervous system, human commensal microbes, and the gut–brain axis and developed five recommendations to increase the likelihood that biotic interventions will lead to meaningful clinical impacts. Interventions should: (1) strive to modulate or refine, rather than “boost,” host metabolism and the immune system to achieve specific outcomes; (2) consider the human metabolome, in addition to the microbiome, as a readout and therapeutic target; (3) determine context-dependent responses to biotics in terms of spatial location within the gut, host genetics, environmental influences, and diet; (4) explore a wide range of microbial fermentation substrates that yield biologically active products in addition to prebiotic fibers; and (5) leverage new approach methods including *ex vivo* human tissue-based approaches, novel *in vitro* methods, and advanced computational tools including artificial intelligence to complement human studies. The roadmap proposed by this panel aims to accelerate the translation of biotic research into discoveries that will more readily impact clinical practice.

## Background

The International Scientific Association for Probiotics and Prebiotics (ISAPP) annual meeting held July 15–17, 2025, in Banff, Canada, included discussion groups of invited experts bringing diverse perspectives to address current challenges in the field and generate consensus regarding important areas for future research. One of these discussion groups aimed to identify potential targets to optimize the host response to biotics. Each of the panel members/authors was chosen for their expertise in a given domain and tasked with reviewing evidence for actionable targets in that domain.

While a variety of biotics have been explored in human trials and in pre-clinical studies with animal models, traditionally, the selection of product, dose, and means of administration seems to owe more to conjecture or convenience than to a rigorous, mechanistic scientific foundation. This approach was not unreasonable when little was known regarding the structure and function of the gut microbiome or of mechanisms by which biotics interact with the host. The thesis that formed the basis of this discussion group was that, given tremendous recent progress in our understanding of the host response, a much more rational and informed process could now guide the precise selection of a biotic for a given indication. To achieve this goal, we explored various mediators and mechanisms of the host response including, but not limited to, components of host immunity and metabolism, neuro-endocrine responses, the commensal intestinal microbiome, and the gut–brain axis. The primary aim of this discussion group was not to provide a comprehensive or systematic review of the biotic literature. Rather, we sought to highlight recent examples of mechanistic discoveries that show promise for future translation into clinical impact. The goal of this meeting summary paper, therefore, was to review the status of targets for biotic interventions and, in so doing, identify validated actionable targets as well as areas where knowledge gaps persist. The latter process would provide directions for future research which, underpinned by a knowledge of the exact biological entity being targeted, lead to more precision and selectivity in the prescription of a biotic in a given clinical situation.

The basic mechanisms and pathways whereby such modulations occur were explored, the accessibility and reproducibility of potential biotic targets critically evaluated, and an attempt to prioritize targets in terms of their relevance to individual biotics and potential for translation to human studies undertaken. Relevant clinical trials were also reviewed and challenges identified. Looking to the future, we discussed emerging approaches for studying host-microbiome interactions (e.g., human-derived organoids, gut-on-a-chip, machine learning approaches). Finally, and mindful of the challenges inherent to translation from bench to bedside in this area, we developed recommendations for the future design of human studies with particular attention to the choice of biotic product and formulation, the baseline diet of the target population, and clinically relevant and measurable human disease outcomes.

## Targets for modulation by biotics

### Target 1: the immune system

The critical role of the microbiome in the development and maintenance of gut- or mucosa-associated lymphoid tissue (GALT or MALT), as well as in the development of immune tolerance, underlines the importance of interactions between the microbiome and the immune system in homeostasis. The ability of the immune system to differentiate between friend and foe has been amply and repeatedly demonstrated through the contrasting immunological responses to a commensal bacterium and a bacterial pathogen, with the former eliciting a regulatory T-cell response associated with anti-inflammatory cytokines and enhanced epithelial barrier function while the latter evokes an inflammatory response mediated by innate and adaptive immune cells requiring activation of a range of intracellular pathways including those regulated by nuclear factor-κB (NF-κB).^1^ Accordingly, probiotics and other microbiome-modulating interventions have been developed to activate host pattern recognition receptors in a manner that induces an anti-inflammatory response.^2^ ^,^ ^3^ Fundamental to this strategy is the detailed characterization of the immunological responses to a given biotic. In the past, the emphasis in research on microbe-host immune responses had been on intact bacteria, for example, the use of *Akkermansia massiliensis* to overcome gut microbiota perturbations that restrain chimeric antigen receptor (CAR) T cell therapy in patients with B cell lymphomas leading to CAR-T cell niching and improved tumor control.^4^

More recently, the immunological properties of bacterial components^5–7^ and, especially, bacterial metabolites, and their role in shaping host immune function is being increasingly recognized, thereby providing novel and attractive mechanisms and targets for biotic interventions.^8^ ^,^ ^9^ These metabolites can be absorbed from the gut and gain access to the circulation where they can exert immunological effects at sites distant from the gut. Immunoregulatory bacterial metabolites can activate host G protein-coupled receptors (GPCRs), aryl hydrocarbon receptors (AhRs), nuclear hormone receptors including the farnesoid X receptor (FXR), or can modulate gene expression through epigenetic mechanisms—all, therefore, targets for biotic interventions. Importantly, many immunoregulatory bacterial metabolites are generated from dietary components (e.g., fiber, amino acids), linking diet and lifestyle to immune and metabolic health via microbial mechanisms. Recent changes in dietary habits and microbiota composition have resulted in reduced levels of those immune regulatory metabolites that are evolutionarily hardwired into immune cell development and decision-making processes.^10–12^ This deficiency of immune regulatory molecules, potentially coupled with an excess of proinflammatory mediators, results in a hypersensitive immune system that reacts inappropriately to non-pathogenic stimuli and does not respond effectively to infection but is associated with a poorly regulated immune system that contributes to increased risk of inflammatory diseases throughout the lifespan.^13–15^

Some of the best described microbial metabolites include short-chain fatty acids (SCFAs), tryptophan derivatives, secondary bile acids (and their derivatives), histamine, sphingolipids, polyamines, and *p*-cresol. This list will, undoubtedly, expand. Bacteria generate SCFAs by fermenting complex carbohydrates such as those typically found in dietary fibers. Intestinal SCFAs include acetate, propionate, butyrate and valerate, which play an important role in intestinal homeostasis.^16^ Multiple GPCRs, including GPR43, GPR41, GPR109A, and LFR78/OR51E2, are activated by SCFAs, while butyrate can also regulate gene transcription via inhibition of histone deacetylases (HDACs). Multiple immune cell populations are impacted by SCFAs initiating an array of regulatory effects that are important for preventing allergies and chronic inflammatory diseases.^17^ ^,^ ^18^ In addition, gut microbiota composition, specifically, colonization with butyrate-producing bacteria, is associated with a reduction in the likelihood of hospitalization for an infectious disease in the general population.^19^

Tryptophan-derived metabolites such as indole, indole-3-lactate, indole-3-propionate (IPA), and indoleacetic acid can be produced by a variety of gut microbes through direct transformation of tryptophan. Many of these metabolites are ligands for AhRs or the pregnane X receptor (PXR). Microbial tryptophan-derived metabolites directly influence epithelial barrier integrity, mucosal lymphocytes, innate lymphoid cells, macrophages, dendritic cells, and neutrophils, thereby reducing inflammation and promoting anti-inflammatory cytokines such as IL-10 and IL-22.^20^ ^,^ ^21^ Levels of IPA, in particular, negatively correlate with a wide range of conditions from COVID-19 cytokine storms, risk of long COVID, and inflammatory bowel diseases.^22–24^ The protective effects of IPA may be mediated via reduced mitochondrial oxidative damage and changes in immune cell metabolism.^25^

Secondary bile acids and their derivatives are generated in the intestine from bacteria-mediated transformation of primary bile acids. These bile acid derivatives can signal in immune cells via multiple receptors including FXR, PXR, liver X receptor, and others.^26^ For example, failure of oral immunotherapy for peanut allergy is associated with distinct patterns of gut microbial bile acid and amino acid metabolism.^27^ Similarly, bacterial-derived histamine is immunologically active and influences both innate and adaptive immune responses via activation of four different GPCRs, the histamine receptors 1-4 (H1R-H4R).^28^ Histamine secretion by microbes in the gut influences mucosal Th2 cytokine responses and reduces allergic inflammation in murine models via the H2R.^29^ ^,^ ^30^ High levels of trace amines (such as tyramine, tryptamine and phenylethylamine) in infants are associated with atopic diseases, including food allergy, which are thought to result from the metabolic activity of Firmicutes including *Tyzzerella nexilis*, *Enterococcus faecalis* and *Ruminococcus gnavus*.^31^ In contrast, production of polyamines such as spermine and spermidine by gut microbes may contribute to regulatory immune responses.^32^ ^,^ ^33^

Arguably, the impact of gut microbes on immune health may be most pronounced during infancy, when lifelong immune activation thresholds and trajectories are established. Disruptions to microbial metabolism can arise from various sources, including antibiotic use, infections, formula feeding, environment, and caesarean rather than vaginal delivery.^34^ Early antibiotic exposure is associated with differences in gut microbial community composition and metabolic activity, impacting the immune system's ability to regulate inflammatory responses, leading to an increased risk of allergic diseases such as asthma and autoimmune disorders.^35^ The avoidance of infections, and consequently antibiotic use, was shown to reduce the risk of food allergy in a cohort of infants born during the enforced social distancing restrictions of the COVID-19 pandemic.^36^ ^,^ ^37^ In addition, close contact with siblings was important for the transfer of non-spore-forming microbes such as bifidobacteria, while Kyoto Encyclopedia of Genes and Genomes (KEGG) orthologs relating to SCFA and tryptophan metabolism were influenced by the presence of siblings with an overall predicted increase in microbial metabolic output.^38^ The “hygiene hypothesis” is based on the observation that children with older siblings had reduced risk of hay fever.^39^ These recent studies suggest an alternative explanation for the “hygiene hypothesis,” whereby transfer of commensal microbes with immunoregulatory metabolic pathways, but not exposure to pathogens, might be responsible for protective effects among siblings. All of these observations illustrate the vulnerability of the developing microbiome as well as the window of opportunity for biotic interventions to address deficits and forestall the later development of illness driven by disturbances to the gut microbiome in early life.

A number of human clinical studies have evaluated the immunomodulatory effects of biotics. For example, oral administration of *Bifidobacterium longum* 35624 reduced circulating C-reactive protein (CRP) and proinflammatory cytokine levels in patients with ulcerative colitis, chronic fatigue syndrome, or psoriasis,^40^ supporting *in vitro* evidence^41–44^ that this bacterial strain induces a systemic regulatory immune response. In addition, a combination of bifidobacterial strains and prebiotics hastened antibody formation against SARS-CoV-2 and alleviated symptoms in patients with long COVID.^45^ ^,^ ^46^ The risk of recurrent respiratory tract infections in children was lower in those administered a lyophilized bacterial extract,^47^ while a fiber supplement reduced CRP levels in pre-diabetic individuals.^48^ These examples highlight the potential for well selected biotic interventions to impact the immune system; however, many human studies fail to recapitulate effects observed in animal models. This may be due to a mismatch between the selected biotic and the immune mechanism being targeted; host-specific factors such as age, pre-existing microbiota composition, or dietary habits; or disease-associated factors such as severity, tissue remodeling, and medication use. Future studies examining the immunological effects of biotics should take these factors into consideration in study design.

### Target 2: host metabolism

Host-microbial interactions regulate metabolic homeostasis, and biotics could help fine-tune energy metabolism to maintain health or to mitigate obesity and cardiometabolic diseases. However, few biotics reproducibly prevent or ameliorate metabolic diseases in human studies or even in relevant animal models of obesity and metabolic syndrome. A search on ClinicalTrials.gov and published studies (PubMed) revealed that more than 150 clinical studies have tested whether defined probiotic strains or combinations thereof modulate intestinal or metabolic endpoints as a primary outcome. These trials tested anywhere from 1 to 14 strains in a single intervention. Most multi-strain probiotics used combinations of *Bifidobacterium* and *Lactobacillus* spp., but in many instances, specific strain designations were not reported and results across trials remain heterogenous. Furthermore, outcomes for clinical interventions often were underpowered, and a one-size-fits-all approach currently seems to lack the precision needed for providing individual benefits.^49^

In only a few well-controlled human trials have probiotics been reported to slightly improve metabolic outcomes and more so, in women who were overweight or obese.^50^ Among probiotics showing promise in human trials are those derived from the genera *Akkermansia*, *Bifidobacterium*, and *Faecalibacterium*. For example, *Akkermansia muciniphila* ATCC BAA-835 reduced inflammation and counteracted metabolic syndrome features including obesity, insulin resistance and liver dysfunction in a proof-of-concept clinical study in overweight and obese adults.^51^ Supplementation with the *B. longum* strain BL21 improved lipid profiles in a randomized controlled trial involving 66 overweight and obese adults.^52^ *Bifidobacterium breve* BBr60 improved serum metabolic profiles and lowered liver transaminases among patients with obesity,^53^ and, in a separate study, increased HDL cholesterol and reduced total cholesterol levels in healthy adults.^54^

Preclinical studies with animal models of obesity and metabolic dysfunction have provided mechanistic insights into how some probiotic bacteria can exert metabolic benefits. Both live and pasteurized *A. muciniphila* have beneficial metabolic effects and the pasteurized bacterium was found to engage Toll-like receptor 2 (TLR2) via its heat-stable outer membrane protein Amuc_1100, revealing a mechanism that leads to suppression of inflammation and improves metabolic features in obese mice.^55^ Another study revealed that live *A. muciniphila* can act through a secreted protein (P9 protein) to stimulate glucagon-like peptide (GLP)-1 secretion and improve glycemic control in obese mice.^56^ One *B. longum* strain (APC1472) was reported to regulate early-life feeding behaviors induced by an unhealthy diet in obese mice with a correction of alterations in tryptophan metabolism. This effect was more evident in adult female mice and appeared to be driven by normalization of behaviors rather than effects on the microbiome.^57^ Also of interest is the finding that *Faecalibacterium prausnitzii* can reduce body weight and inflammation and can improve gut barrier integrity in association with enhanced butyrate production in obese mice.^58^

To advance the field, relevant models of human metabolic disease should be used in thoughtfully designed preclinical studies that carefully consider critical aspects including strain, dose, and mode of administration of the biotic, appropriate power and sample size calculations *a priori*, and thoughtful attention to potential confounders including environmental features (e.g. single vs co-housing, bedding/temperature, diet, baseline microbiome differences) that can influence the gut microbiome and host responses.^59^ ^,^ ^60^ Many recent animal studies testing biotics are still designed to capture large effect sizes, akin to trials testing diets that differ markedly in multiple key nutrients, or they are designed in a manner similar to that employed in trials of pharmaceuticals that act via a single robust mechanistic target. However, immediate and robust changes in gut microbiota composition and host metabolism are difficult to achieve. Biotics are not drugs; they target host-microbial interactions in more subtle ways and generally act to influence metabolism by fine-tuning immune-metabolic processes. Therefore, effect sizes of biotics on metabolic parameters are predictably more modest, thus the need to administer a biotic for a longer duration and/or through study designs which are adequately powered to detect the desired effects. The importance of careful selection of biological endpoints and disease outcomes cannot be understated.

Perhaps the greatest confounder in biotic research, especially when examining the effects of biotics on metabolic endpoints, is diet. This is expected, given the documented impacts of food patterns, diets, and many nutrients on the microbiome and immune-metabolic regulation. Recent publications have highlighted how diet can influence the response to probiotics. Larsen et al.^61^ compared the effects of two lactic acid bacteria (LAB), namely *Limosilactobacillus reuteri* and *Lacticaseibacillus paracasei* subsp. *paracasei*, and reported an internally reproducible yet dichotomous response when two different obesogenic diets were administered for 3 months to produce different degrees of metabolic disease severity. *L. paracasei* administration reduced weight gain, insulin resistance, and hepatic steatosis in mice fed a commonly used high-fat diet (HFD) but not a fast-food mimicking diet (FFMD), the latter causing more obesity, fatty liver disease and more severe diabetic features. Conversely, *L. reuteri* exerted protective effects on diabetes and liver outcomes only in the severe FFMD model. These diet-specific effects were strongly associated with changes in gut microbiota composition and function as well as with the profile of bile acids in the distal gut. Diet-specific impacts on probiotic efficacy also have been studied in humans. Wastyk et al.^62^ reported that a probiotic supplement containing three bacterial strains (*L*. *reuteri* NCIMB 30242, *Lactiplantibacillus plantarum* UALp-05, and *Bifidobacterium animalis* subsp. *lactis* B420) given for 18 weeks to 39 adults with central obesity did not significantly improve the metabolic syndrome. However, significant improvements in blood pressure and lipid profiles were observed in a subset of participants that had a distinct microbiome profile relative to non-responders. Interestingly, baseline diet—and, in particular, consumption of total and added sugar—were key factors in differentiating between responders and non-responders.

The impact of diet on probiotic efficacy also was recently demonstrated in a study of healthy adults^63^ in which a diet mimicking non-industrialized dietary patterns was shown to enhance the intestinal persistence of *L. reuteri*, a bacterium rarely found in industrialized gut microbiomes.^64^ This finding suggests new opportunities to develop targeted probiotic therapies with bacterial strains that are absent or under-represented in industrialized microbiomes (such as *L. reuteri*) and that could contribute missing functions to maintain gut and metabolic health. Prebiotics could also be designed to increase the abundance of these beneficial microbes and thus represent powerful tools for restoring microbiome balance and addressing cardiometabolic diseases and other chronic conditions linked to industrialization.^65^ This study also highlights the importance of considering new opportunities for more personalized, microbiome-targeted dietary interventions aimed at improving long-term health outcomes. Indeed, it is likely that a personalized approach towards the use of probiotics (and prebiotics) in targeting chronic diseases will be required, given the interindividual variations in the response to current probiotic strains and prebiotic products.

Additionally, we must expand the menu of probiotic therapies beyond the few bacterial groups that are most often studied (e.g., LAB and bifidobacteria) and explore other potentially beneficial microbes possessing novel functions relevant to metabolic homeostasis. New metabolic activities of probiotics beyond traditional functions (e.g., SCFA production) must be identified in next-generation candidates, as recently reviewed elsewhere^66–68^ and discussed in the previous section on immune function. In one recent example, the LAB strains *Lactobacillus delbrueckii* subsp. *bulgaricus* CNCM I-1519 and *Streptococcus thermophilus* CNCM I-1630 (typically used to make yogurt) can produce branched-chain hydroxy acids that are derivates of branched chain amino acids, typically only found in fermented dairy products.^69^ These bacterial metabolites are associated with improved glucose regulation and protection from insulin resistance, type 2 diabetes, and fatty liver disease in mice fed a high-fat, high-sucrose diet, and they also directly control glucose metabolism in liver and muscle cells.

### Target 3: the enteric nervous system and gut neuro-endocrine cells

It is well established that gut microbiota play key roles in regulating intestinal physiology, including motility, visceral sensitivity and barrier function, all resulting from complex interactions between the neural, myogenic, immune, and endocrine systems.^70–73^ Studies in gnotobiotic mice demonstrate that absence of microbiota is associated with slower gastrointestinal transit, which normalizes after microbial colonization, with individual bacterial strains having specific effects on intestinal motility.^74^ Although the vast majority of gut bacteria reside in the colon, the effect of microbial colonization on motility is most evident in the small intestine, with changes in the enteric nervous system that include altered regulation of acetylcholine release through vasoactive intestinal peptide-containing neurons at the myenteric plexus.^75^ This interaction between the microbiota and host is highly dynamic, reacting to changes in the composition of the gut microbiota, and is dependent on the innate immune system and enteric glial cells.^75^ Similarly, intestinal permeability and mucus structure is abnormal in germ-free mice and normalizes within one week after bacterial colonization, accompanied by changes in innate and adaptive immunity and decreased systemic bacterial antigen exposure.^76^ ^,^ ^77^ The intestinal neuroendocrine system is also affected, as gut microbiota, especially spore-forming bacteria, can upregulate serotonin biosynthesis from colonic enterochromaffin cells, with corresponding changes in intestinal physiology.^78^ Similarly, visceral sensitivity is increased in germ-free mice, in part due to higher production of calcitonin-gene-related peptide by the gut sensory neurons as well as changes in brain structure; these changes also normalize after bacterial colonization.^79^ ^,^ ^80^

Gut microbes interact with the host through multiple mechanisms, many of which likely differ along the length of the small and large bowel. Though the greatest density of bacteria is found in the colon,^81^ ^,^ ^82^ typically gut immune cells including mast cells, eosinophils, Paneth cells, dendritic cells and intraepithelial lymphocytes are most abundant in the small intestine.^81^ Thus, it likely that microbiota-host immune cross-talk is more pronounced in the small intestine, where the expression of multiple genes related to gut barrier function, motility, sensitivity, and immunity in response to colonization with commensal microbiota has been shown.^75^ Indeed, the small intestine is responsible for digestion and absorption of most nutrients, and thus is exposed to myriad dietary antigens and diet-derived bacterial metabolites.^81^ Site specificity also has been observed in mice colonized with human microbiota. Mice colonized by microbiota from patients with irritable bowel syndrome (IBS) express multiple altered transcripts related to pain signaling, motility and permeability. This set of genes also differs based on the patient donor, suggestive of heterogenous pathophysiology of IBS (*unpublished—Bercik, personal communication*). In that regard, several studies identified specific mechanisms by which gut bacteria impact IBS symptoms, including regulation of host proteases by commensal microbes,^83^ reduced production of bacterial antinociceptive lipids,^84^ sensitization to dietary antigens mediated by infection or bacterial toxins resulting in gut-restricted production of IgE,^85^ production of bacterial histamine by metabolizing dietary histidine resulting in mast cell hyperplasia,^86^ and mast cell activation by bacterial lipopolysaccharide.^87^ ^,^ ^88^

Probiotics and prebiotics have the potential to prevent and reverse the negative effects of the microbiota on the gut neuroimmune system, whether by a generalized modulation of the intestinal immune system or by targeting specific pathways.^89^ Indeed, probiotic bacteria and prebiotics have been shown to be beneficial in some patients with IBS.^90^ ^,^ ^91^ The results of clinical trials reveal high heterogeneity, in part due to differences in study population, trial designs and outcomes, and multiple treatments being tested.^92^ Future trials should be designed to target specific pathways in patient subpopulations selected based on disease pathophysiology, taking into account site-specific differences within the digestive tract.

### Target 4: human commensal microbes

At birth, the gastrointestinal tract is sterile; bacteria immediately colonize the gut and increase rapidly in numbers and diversity over the next 2–3 y. Thereafter, and assuming no disruptions occur, the microbiome remains relatively stable until later life when overall diversity and genera such as *Bifidobacterium* decline. This is not to say that the adult microbiome does not change; it does, but this is best visualized as a state of dynamic equilibrium with some core taxa (keystone species) remaining stable and other taxa fluctuating in the short term.^93^

When disturbed, the microbiota has, in general, a considerable capacity to restore itself, a property referred to as resilience, defined as “the overall capacity of a microbial community to resist or recover from perturbations to its original state of balance.” Commonly encountered perturbations include enteric infections, use of antibiotics and other medications, and dramatic dietary changes.^94^ Resilience does not necessarily equate with health; the acquisition of an unhealthy microbiome (perhaps in the aftermath of one of the aforementioned disruptions) with high resilience might contribute to chronic disease. In contemplating how a biotic intervention might impact microbe-microbiome interactions, it is worth considering what factors contribute to resilience. Before doing so, it is important to concede that most of the available information is derived from studies on bacteria and archaea and tends to ignore the possible contributions of viruses (such as bacteriophages)^95^ and eukaryotes.

In general, a diverse microbiome with functional redundancy will be more resilient. In this and other ways, the status of the pre-perturbation microbiome has a considerable bearing on its subsequent recovery. How do all the species and strains known to inhabit the gut manage to persist? Several mechanisms which promote survival have been identified. Resistance to colonization by a novel strain or to invasion following exposure to an antibiotic is conferred by multiple bacterial and host factors, such as: ability to form biofilms, availability of and access to nutrients, bacterial metabolites (e.g., impact of a lower pH produced by short-chain fatty acids), the mucus layer incorporating various anti-microbial peptides produced by the host epithelium, and immune adaptation (tolerance or loss thereof). Bearing these phenomena in mind, several levels of microbe-microbe interaction have been identified.^94^ ^,^ ^96^ Mutualism or symbiosis (also referred to as cooperation) describes a situation in which microorganisms share resources, coordinate metabolic resources, or share genetic information to mutual benefit; these interactions promote stability. Cross-feeding or syntropy (so-called metabolic symbiosis) refers to the sharing of metabolites by different species, or in the case of archaea, to utilizing hydrogen generated by bacteria to synthesize methane, by different domains. Quorum sensing is a group activity whereby a community of organisms respond to changes in population density, composition and diffusion conditions by communication between bacteria to impact growth, metabolic activity, gene expression or cooperation in the community. Commensalism refers to the coexistence of different microbial species within the same ecological environment but without any exchange of energy or materials between them. Competition involves just what it says, competition between microorganisms for nutrients and space. Amensalism or antagonism involves the production of substances that prevent or limit the growth of other organisms. Examples here include the production of bacteriocins against other strains (e.g., thuricin against *Clostridiodes difficile*)^97^ or of metabolites that change the environment to inhibit or kill other bacteria.

How do biotics cope with such resistance to colonization? The simple answer is that most don't. Most probiotics do not engraft and will disappear from stool not long after supplementation ceases,^98^ their effects, therefore, being unlikely to have been exerted via a change in the composition of the resident microbiome.^99^ Studies examining the impact of probiotics on the gut microbiome in the aftermath of antibiotic exposure failed to identify changes in microbiota diversity with changes in composition being variable,^100^ with one study actually demonstrating an inhibition of microbiome recovery in the presence of certain probiotics.^101^ This is not to say that microbe-microbe interactions are not relevant to probiotic benefits but rather suggest that they are more likely to be effected via more nuanced impacts on bacterial function and metabolism rather than replacement or engraftment.

The microbiota has long been implicated in colonization resistance against pathogens. Infections by pathogens shape this process, and the gut microbiota from previously infected hosts display enhanced resistance. This is due in part to alterations in bile acid metabolism leading to the expansion of taxa that utilize the sulfonic amino acid, taurine. Administration of exogenous taurine alone is sufficient to drive this increase in taurine-utilizing microbes and enhance resistance. Taurine enhances the microbiota's production of sulfide, an inhibitor of cellular respiration, which is key to host invasion by numerous pathogens. This offers a potential prebiotic intervention that is dependent on microbiota modulation of host-derived factors to enhance colonization resistance to subsequent infection.^102^

Diet shapes gut microbial communities, and this could explain why a ketogenic diet (KD) was found to have protective effects on multiple sclerosis, providing novel mechanistic targets for biotic interventions. In a murine model of multiple sclerosis known as experimental autoimmune encephalomyelitis (EAE), KD ameliorated disease in a microbiota-dependent fashion via supplementation of a single KD-dependent host metabolite (β-hydroxybutyrate [βHB]). Transplantation of the βHB-shaped gut microbiota was protective, and findings were recapitulated either by administering indole lactate or by introducing the *Ligilactobacillus murinus* strain that produces indole lactate. In this example, diet alters the gut microbiota to modulate the immune system, integrating diet-host-microbiome interactions.^103^

Recent studies have shed new light onto mechanisms that drive microbe-microbe interactions. Predictive metabolic modeling reveals that loss of species from gut microbial communities, or extinction, can be reversed in many cases by adding back the missing bacteria; microbial seeding effectively restores communities to their original state, but only when the nutrient niche of the lost species has gone unused by competing microbes.^104^ Indeed, long-term extinction of specific gut microbial taxa was documented recently in humans administered not only antibiotics but a surprisingly wide variety of medications.^105^ In a mouse model, seeding via autologous fecal microbiota transfer (FMT) was unable to restore antibiotic-induced perturbations to gut microbial communities unless the diet was enriched in complex carbohydrates to facilitate microbial community feeding networks.^106^ Leveraging machine learning and metabolic modeling, these studies highlight the intestine's nutrient niche as a critical factor that determines how the commensal microbiota responds to challenges. Microbial community-scale metabolic modeling also shows promise predicting how biotic therapies might impact an individual's blood-derived clinical chemistry profile, which could help tailor personalized diet-biotic interventions to improve human health.^107^

The commensal microbiota has also emerged as a therapeutic target in childhood malnutrition. After initial studies revealed that fecal microbial communities from children with the severe, edematous form of malnutrition known as kwashiorkor could produce marked weight loss when transplanted into recipient gnotobiotic mice,^108^ a microbiota-directed complementary food (MDCF) was designed to support weaning-phase gut bacterial species that were depleted in malnourished children.^109^ After 3 months, the MDCF increased the abundance of growth-associated bacteria and modestly improved weight-for-length z scores in Bangladeshi children with moderate acute malnutrition^110^ or severe acute malnutrition.^111^ Mechanisms by which targeting the commensal microbiome can improve child growth are being elucidated. Two prebiotic carbohydrate components of the MDCF are selectively utilized by growth-associated gut bacteria. These include prebiotic glycan metabolized by *Prevotella copri* strains that contain specific polysaccharide-utilization loci^112^ ^,^ ^113^ and prebiotic glucomannan which is metabolized by *Segatella copri* strains containing specific carbohydrate-active enzymes (CAZymes).^114^ Prebiotic carbohydrates are not the only microbiome-targeting components of the MDCF that can improve growth. A fatty acid amide hydrolase in a growth-associated strain of *F. prausnitzii* can synthesize a variety of N-acylamides including amino acids, which could have numerous beneficial effects on child growth.^115^

### Target 5: the microbe–gut–brain axis

The concept of the gut–brain axis^116^ has proven valuable in understanding gastrointestinal symptomatology and dysfunction in neurological disorders such as Parkinson's disease and multiple sclerosis, as well as in enabling patients and their physicians to understand how stress, as well as psychological disorders such as anxiety and depression, can initiate or exacerbate gastrointestinal symptoms. With the recognition that the microbiome can participate in the cross-talk, the concept of the microbiome–gut–brain axis emerged.^117^ Indeed, that the gut microbiome can influence brain function was demonstrated many years ago in studies that revealed how bacterial metabolism of proteins led to the generation of ammonia and other neurotoxins resulting in hepatic encephalopathy in individuals with chronic liver disease.^118^ More recently, Braak hypothesized, first, that the pathology that results in the clinical features of Parkinson's disease ascends from the lower brainstem and, second, that this ascending migration begins with the entry of a pathogen through the nose to the olfactory bulb and then, when swallowed, enters the gastrointestinal tract, gains access to the enteric nervous system and initiates the aggregation of α-synuclein*.* Eventually, aggregated *α*-synuclein spreads towards the central nervous system via the vagus nerve.^119–121^ While this hypothesis has not gained universal acceptance, it does provide a framework to explain how a microbe could influence brain function, a concept that has been applied to other areas. Indeed, evidence has accumulated from a large volume of experimental data in animal models to illustrate how the microbiome can influence brain structure and function, changes that translate into altered behavior.^122^ ^,^ ^123^

In addition to being variably associated with diverse neuropsychological diseases, the gut microbiome has also been shown to influence substance use disorders (SUDs). Unravelling mechanistic interactions between gut microbes and the host during psychostimulant use remains challenging. Cocaine exposure increases intestinal levels of norepinephrine, sensed through the bacterial adrenergic receptor QseC to promote intestinal colonization of γ-Proteobacteria.^124^ Metabolomic, metagenomic and transcriptomic studies showed that this shift in microbiota composition depletes the neuroactive metabolite glycine (used as a nitrogen source by γ-Proteobacteria) in the gut and cerebrospinal fluid, enhancing host cocaine-induced behaviors. Glycine repletion reversed this response, and intestinal colonization by γ-Proteobacteria unable to uptake glycine did not alter the host response to cocaine.^124^ Glycine is synthesized from sarcosine, which is also depleted during γ-Proteobacteria gut expansion, because mammalian cells are using it to synthesize glycine. Importantly, sarcosine repletion also mitigates the enhancement of cocaine-mediated behaviors. Sarcosine is commonly used by athletes as a nutritional supplement, tying in dietary modifications with mitigation of SUDs. Transcriptomic profiling indicates a role for microbiota-modulated glycine levels in cocaine-induced transcriptional plasticity in the nucleus accumbens through the glutamatergic transmission. Altogether, we introduce a mechanism by which intestinal bacteria alter the host's brain responses to cocaine that could be exploited to modulate reward-related brain circuits that contribute to SUDs.^124^

More challenging has been the demonstration of how the microbiome–gut–brain axis is operative in humans. Stress responses have provided an opportunity to explore this axis in human subjects.^125^ Here, gut microbiome modulation by a probiotic ameliorated the stress response.^126–128^ In IBS, a chronic, recurrent disorder characterized by pain and altered bowel habit, widely considered as an exemplar of the bidirectionality of the gut–brain axis, microbiome modulation with probiotics has been shown to alleviate gastrointestinal symptoms.^91^ ^,^ ^129^ Meta-analyses have shown that probiotics in aggregate and *Escherichia* and *Lactobacillus* strains as well as multi-strain preparations individually, can be effective.^91^ While studies with other biotics are fewer in number, well-conducted studies have also demonstrated efficacy for at least one postbiotic,^130^ prebiotic,^131^ and synbiotic^132^ preparation in IBS. Probiotics have also been shown to ameliorate common co-morbidities in IBS such as anxiety and depression.^133–135^ Indeed, there is some, albeit far from conclusive, evidence that probiotics may have a positive impact on anxiety and depression, in general.^136–138^

FMT, a complex microbial intervention, has shown some hints of efficacy in Parkinson's disease,^139^ depression,^140^ and IBS.^141^ One must caution, however, that results in any of these contexts have not been consistent and, except for a proposed role for butyrate in the efficacy of FMT in IBS,^142^ mechanism(s) of action is/are unknown. Differences in the nature of the delivered product, its mode of delivery, and study protocol also complicated interpretation. These reservations notwithstanding, the ability of FMT to modulate central nervous system functions, including mood, has prompted a search for the microbial elements that are essential to confer efficacy in any given disorder—we may have set out on the road to personalized bacteriotherapy, but there is a really long way to go and many barriers to overcome.

## Novel concepts emerging from these discussions

In keeping with current trends in microbiome research, in general, we foresee a greater emphasis on microbiome metabolites, microbiome-microbiome and microbiome-host interactions in the future and less on microbiome composition or relative “numbers.” A similar shift has taken place in developing outcome measures for clinical trials of biotics with the past obsession with simply adding in more microbes to an emerging emphasis on subtle and nuanced effects on the commensal microbiome and the host. For example, immunological effects should be evaluated in terms of their ability to modulate the immune response and not simply as being anti-inflammatory. Perhaps, in part, related to a perceived regulatory advantage, prebiotics and postbiotics are enjoying increasing research and clinical interest with specificity in relation to the former and product optimization for the latter being important future goals. The well-documented success of FMT in very specific clinical scenarios has generated interest, on the one hand, in the use of bacterial consortia and, on the other, in attempting to define what components (and not just bacteria!) in an FMT are truly responsible for its efficacy. The influence of the donor on success in a number of areas where FMT has experienced more variable responses provides further impetus to identifying the donor and recipient characteristics that predict success. Beyond FMT, hints are beginning to emerge that host phenotype (and, perhaps, genotype) may be an important component of the response to biotics and here we are alluding not just to the host microbiome but to other demographic, psychosocial and biological traits—undoubtedly, an area ripe for productive research. At-home microbiome testing kits are already widely available, though they are scarcely supported by high-quality evidence for their impact on clinical outcomes. This could change and a “test and treat” approach ultimately validated whereby an individual's baseline microbiome and personal and dietary data are combined to select the optimal biotic(s). These developments have fundamental implications for product development and the design of future clinical trials.

## How to optimize the host response to biotics?

Any attempt to develop a biotic intervention targeting one or more of these five human systems must confront several important challenges. We discuss these challenges in the context of biotics that target the gut–brain axis.

### 1. Subject selection

Currently, we have little to guide us on who is most likely to respond to a biotic. Ideally, one should define a phenotype most receptive to biotic intervention based on genetics, demographic features, and disease characteristics (e.g., subtype, severity, level of disease activity), but such information is rarely available. From data in other fields, it has become clear that the baseline microbiome could be an important determinant of treatment response.^143–145^

### 2. The intervention

Ideally, any biotic intervention should be tailored to a specific disease or specific populations. Achieving such specificity demands a lot more knowledge than we currently have. Available data on biotic interventions in relation to the gut–brain axis are limited and rife with problems. How will the intervention mediate its effect? It is a long way from the gut lumen to the brain, and many obstacles lie in the path. Does the biotic exert its effects through engagement with the immune system? This is certainly plausible given the evidence supporting immune-modulating effects of the microbiome^146^ and biotic interventions, such as probiotics^2^ ^,^ ^20^ on the brain. An anti-inflammatory approach might well be relevant to Alzheimer's disease where neuro-inflammation is thought to be an important factor.^147^ A relevant example of an anti-inflammatory mode of action comes from a study showing that improvements in mood in IBS were associated with parallel declines in levels of tumor necrosis factor alpha.^40^ Microglia have recently emerged as another potential target for interventions along the microbiome–gut–brain axis.^148^ Does the biotic act through the synthesis and release of neuromodulatory peptides?^79^ ^,^ ^86^ The observation that histamine and calcitonin gene-related peptide can modulate visceral sensitivity through effects on visceral afferents provides one targetable mechanism of action.^79^ ^,^ ^86^ Alternatively, do bacterial components or metabolites traverse the gut barrier and gain access to the submucosal compartment and then on to the circulation? While the ability of *Salmonella* to do so has been demonstrated,^149^ and bacterial fragments contained within intestinal dendritic cells have been shown to reach the mouse brain,^150^ we have yet to see evidence that a beneficial bacterium can do likewise. There is experimental^151^ and clinical^134^ evidence to suggest that bacteriotherapy can modulate the hypothalamic-pituitary-adrenal axis, another potential therapeutic target. If the goal of therapy is to modulate the microbiome, reverse “dysbiosis,” or repopulate deficient species, one requires a thorough understanding of the baseline microbiome and how it leads to the expression of the disease state. Despite a plethora of studies describing various alterations in the gut microbiome in these diseases, a characteristic and consistent signature has yet to emerge here, as in many other disease states in which the microbiome has been implicated. Progress seems likely as some disease-related microbiome and metabolome patterns linked to disease mechanisms^152^ ^,^ ^153^ and even causative factors^154^ have begun to emerge in Parkinson's disease, for example. A greater understanding of just how the microbiome interacts with the gut–brain axis will also dictate issues that will be generic to any biotic intervention: formulation, dose, mode of delivery, and benefits of co-therapies such as diet and prebiotics.

### 3. The outcomes

The interpretation of probiotic studies is often marred by a lack of consistency in outcomes. Whether designed to prevent or treat a given condition, a biotic intervention study must have clearly defined outcomes which may range from primary prevention to cure to alleviation of symptoms. In relation to disorders of gut–brain interaction, these outcomes will be largely symptom-based and should employ validated instruments which track not just symptom responses, but also more global endpoints such as measures of quality of life. For neurodegenerative disorders such as Parkinson's disease, widely used and validated scales that combine cardinal symptoms with functional status and mobility are available. “Hard” endpoints are more difficult in this area, but advanced brain imaging has already shown promise in being able to detect probiotic-induced changes.^127^ ^,^ ^133^

### 4. Identifying therapeutically actionable targets

In the annals of the gut–brain axis, the concept of the microbiome–gut–brain axis is a relatively recent chapter. Almost all data on how the gut microbiome impacts the gut brain axis has been derived from animal models which incompletely recapitulate the human condition and where phenomena such as mood are impossible to capture. Such studies have demonstrated that the gut microbiome and biotic interventions can influence brain structure and function and modify behaviors. These observations kindled much expectation for the application of “psychobiotics” and other biotic interventions to human disease. The reality is much more complex. Progress has been made and some potentially actionable targets along the gut–brain axis have been identified, but much more needs to be done. Translational work on microbiome–gut–brain axis communication should permit a more precise delineation of those pathways that are most relevant to a disease process and, thereby, isolate the best targets for intervention. Meanwhile, large scale, longitudinal multi-omics studies of well-phenotyped subjects matched with appropriate controls should provide a more thorough understanding of how microbial changes relate to disease expression and progression. An appropriate biotic intervention can then be designed and tested.

### 5. Translation to practice

The biotic literature is replete with examples of biotics that worked wonders in animal models (Table 1) only to fail dismally in human studies. Understanding the limitations of animal models while at same time optimizing the translatability of any given pre-clinical strategy will be critical—make your pre-clinical model as relevant as possible to the human problem. Too many clinical trials of biotics suffer from fundamental flaws in clinical design; we emphasize the need to prioritize appropriately powered and designed studies with relevant comparators and meaningful outcomes. This essentially moves biotics into the drug arena with its attendant costs. For example, more studies of live biotherapeutic products (LBPs, as probiotics designed to address disease claims are now referred to by the United States Food and Drug Administration) will be needed, but who will pay for such studies? Successful products emerging from such high-quality pivotal trials will be priced at a premium; given the relatively modest therapeutic gains for most biotics (Table 2), will the investment be recouped, and what will be the implications for the individual consumer or health care systems? Currently largely embedded in the food supplement (low cost, high cost, low return) category, there has been little impetus for manufacturers to invest heavily in clinical trials. The regulatory climate may change and demand more rigor in relation to clinical claims, be they precise or vague. In Europe, the European Food Safety Authority (EFSA) already regards the use of the term “probiotic” as an implied health claim. While safety has, for the most part, not been a concern for healthy individuals, the occasional report of serious infection in relation to probiotic use may offer a regulatory advantage to products such as prebiotics or postbiotics that do not contain live organisms. Though demonstrating exciting promise in the laboratory, regulatory issues may preclude further progress in genetically modified organisms.

**Table 1.** Key findings from preclinical models—mechanisms underlying the host response to biotics.

Host target	Biotic	Model	Mechanism	Ref
Immune system	Exopolysaccharide purified from *B. longum* 35624	Mouse model of ovalbumin respiratory allergy	Reduce eosinophil recruitment via TLR2 mediated IL-10 secretion	[6]
	Cell wall isolated from *B. longum* 35624	Mouse model of influenza infection	Reduce viral load and protect against virus-induced inflammatory sequelae	[7]
	*A. massiliensis*	Syngeneic mouse CAR T cell tumor model	Induce CAR T cell niching and Tc1 CD8+ T cell differentiation, promoting tumor control in AhR-dependent manner	[4]
Metabolism	*A. muciniphila* (live and pasteurized)	Obese and diabetic mice	Engage TLR2 via the outer membrane protein Amuc_1100	[55]
	*A. muciniphila*	Obese mice	Secreted P9 protein promotes GLP-1 secretion and regulation of blood sugar	[56]
	*B. longum* APC1472	Obese mice	Restore feeding behavior	[57]
	*F. prausnitzii*	Obese mice	Confer anti-obesity and anti-inflammatory effects, enhance gut barrier function possibly via butyrate production	[58]
Enteric nervous system and gut neuro-endocrine cells	Lactic acid produced by LAB	Mouse model of IBS	Decrease histamine production by inhibiting the pH-sensitive bacterial enzyme, histidine decarboxylase	[86]
	Metabolites from spore-forming microbes	Germ-free mice	Promote serotonin synthesis by enterochromaffin cells to increase gut motility	[78]
Commensal microbes	Thuricin CD, a bacteriocin produced by *Bacillus thuringiensis* DPC 6431	Bacterial culture overlays (zone of inhibition)	Post-translational modifications allow peptides Trn-alpha and Trn-beta to act synergistically to kill *Clostridioides difficile* with little impact on most other genera	[97]
	Taurine	Mice previously infected with a food-borne pathogen	Potentiate the microbiota's production of sulfide, an inhibitor of cellular respiration, thereby promoting resistance to subsequent infections	[102]
Microbe–gut–brain axis	Various probiotics (e.g. *Lactobacillus*, *Bifidobacterium*, *Blautia*)	Various models of animal behavior and stress responses	Modulate central nervous system development and function, down-regulate stress responses and anxiety- and depression-like behaviors	[125]

**Table 2.** Illustrative selected findings from human studies.

Human target	Biotic	Population	Key finding	Ref
Immune system	*B. longum* 35624 at 1 × 10^10^ cfu/d for 6 or 8 weeks	Adults with ulcerative colitis (*N* = 22), chronic fatigue syndrome (*N* = 48), or psoriasis (*N* = 26)	Reduced circulating CRP and proinflammatory cytokines	[40]
	Commercially available synbiotic combination of *Bifidobacterium adolescentis*, *Bifidobacterium bifidum*, and *B. longum* (1 × 10^11^ cfu/d), galactooligosaccharides, xylooligosaccharides, and resistant dextrin for 28 d	Adults hospitalized with COVID-19 (*N* = 25)	Increased antibody formation against SARS-CoV-2, reduced nasopharyngeal viral load, reduced pro-inflammatory immune markers, and altered gut microbiota vs non-randomized controls	[45]
	Commercially available synbiotic combination of *B. adolescentis*, *B. bifidum*, and *B. longum* (2 × 10^10^ cfu/d), galactooligosaccharides, xylooligosaccharides, and resistant dextrin for 6 months	Adults with post-acute COVID-19 syndrome (PACS; *N* = 463)	Alleviated multiple PACS symptoms vs placebo, with implications for other post-infectious conditions	[46]
	Fiber blend of 56% prebiotic fiber (11.2 g) and 44% viscous/low-fermentable fibers (8.8 g) daily for 16 weeks	Adults with a primary diagnosis of pre-diabetes (*N* = 66)	Improved insulin sensitivity and CRP levels vs placebo	[48]
Metabolism	Live or pasteurized *A. muciniphila* ATCC BAA-835 10^10^ bacteria per day for 3 months	Overweight and obese individuals (*N* = 40)	Reduced inflammation, obesity, insulin resistance and markers of liver dysfunction	[51]
	*B. longum* BL21 2 × 10^10^ cfu/d with 3 g maltodextrin for 8 weeks	Overweight and obese individuals (*N* = 66)	Improved lipid profiles	[52]
	*B. breve* BBr60 1 × 10^10^ cfu/d for 12 weeks	Obese individuals (*N* = 75)	Improved serum metabolic and liver enzyme profiles	[53]
	*B. breve* BBr60 1 × 10^10^ cfu/d for 8 weeks	Healthy adults (*N* = 109)	Increased HDL cholesterol and reduced total cholesterol levels	[54]
Enteric nervous system and gut neuro-endocrine cells	Systematic review of specific strains or combinations of probiotics	Adults with IBS (*N* = 10,332 in 82 trials)	Global symptoms improved by *Escherichia* spp., *Lactobacillus* spp., *L. plantarum* 299V, and combination probiotics; abdominal pain improved by *Saccharomyces cerevisae* I-3856, *Bifidobacterium* spp., and combination probiotics; abdominal bloating or distension improved by *Bacillus* spp. and combination probiotics	[91]
	Systematic review of various strains or combinations of probiotics	Adults with IBS (*N* = 5545 in 53 trials)	Certain species and strains or combinations of probiotics had beneficial effects on global IBS symptoms and abdominal pain	[90]
Commensal microbes	*B. longum* 35624 at 1 × 10^9^ cfu/d for 8 weeks	Adults with IBS who received either encapsulated oral probiotic (*N* = 39) or placebo (*N* = 37), and healthy adults who received the probiotic (*N* = 41)	Transiently increased fecal *B. longum* 35624 in healthy adults and those with IBS, with limited impact on 10 other taxa and no impact on IBS symptoms	[98]
	Combination of 11 strains of *B. bifidum, L. rhamnosus, L. lactis, L. casei* subsp*. casei, B. breve, S. thermophilus, B. longum* subsp*. longum, L. casei* subsp*. paracasei, L. plantarum,* and *B. longum* subsp*. infantis,* at least 25 billion bacteria twice daily for 28 d	Healthy adults given oral broad-spectrum antibiotics for 7 d followed by probiotics (*N* = 8) or no treatment (*N* = 7)	Probiotics delayed and incompletely restored pre-treatment stool and mucosal microbiome	[101]
	Microbiota-directed complementary food prototype-2 (MDCF-2; containing chickpea flour, peanut flour, soybean flour and green banana), which supports growth-discriminatory gut bacteria in preclinical models, twice daily for 3 months	Bangladeshi children with moderate acute malnutrition receiving MDCF-2 (*N* = 59) or standard therapeutic feeds (*N* = 59)	MDCF-2 yielded modest improvements in rates of change in weight-for-length Z-score, weight-for-age Z-score, and mid upper arm circumference	[110]
Microbe–gut–brain axis	*B. longum* NCC3001 at 1 × 10^9^ cfu/d for 6 weeks	Adults with IBS receiving probiotic (*n* = 22) or placebo (*n* = 22)	Improved comorbid depression and IBS symptoms, altered brain activation patterns on functional magnetic resonance imaging	[133]
	*B. longum* 1714 at 1 × 10^9^ cfu/d for 4 or 8 weeks	Healthy volunteers exposed to stress (*N* = 20 to 40 per study)	Short-term reduction in stress responses	[126– 128]
	*B. longum* NCC3001 at 1 × 10^10^ cfu/d for 6 weeks or the combination of *B. longum* 35624 and 1714 at 1 × 10^9^ cfu/d for 8 weeks	Adults with IBS and co-morbid anxiety and/or depression (*N* = 35 or 40)	Reduction of IBS symptoms	[134, 135]

## Recommendations for the future

As the discussion group reviewed the current state of knowledge regarding interactions between biotics, the host, and the gut microbiome, recurring themes emerged. The group of experts proposed the following five recommendations, which were developed during the face-to-face meeting of the group and unanimously agreed upon and later circulated via email for any final comment or revisions, to accelerate the path to meaningful clinical impacts for biotics (Table 3).

**Table 3.** Recommendations to increase the likelihood that future biotic interventions will lead to meaningful clinical impacts.

1. Aim to modulate or refine, rather than “boost,” host immunity and metabolism.
2. Consider the human metabolome, in addition to the microbiome, as a readout and therapeutic target.
3. Determine specific context-dependent responses to biotics.
4. Explore a wide range of bioactive microbial metabolites (e.g., not just fermentation of dietary polysaccharides into SCFAs).
5. Leverage new approach methods (NAMs) to increase predictive accuracy for human health outcomes.

**Recommendation 1: aim to modulate or refine, rather than “boost,” host immunity and metabolism**. We should avoid trying to make biotics “boost,” but rather aim to fine-tune or modulate, complex human systems. Host metabolism and immunity are significantly more complex than a muscle that can be strengthened with training. Therapies should aim to target specific mechanistic pathways within these systems that are likely to impact human health outcomes.

**Recommendation 2: consider the human metabolome, in addition to the microbiome, as a readout and therapeutic target.** Assessing functional potential inferred by DNA sequencing is less expensive, but also less informative, than quantifying the microbial metabolites that mediate biological responses. We must strive to understand how gut microbes influence the human metabolome, and how the metabolome in turn influences specific cells and tissues. Because a large portion of mass spectrometry data is not yet mapped to metabolites, especially those derived from gut bacterial metabolism with unknown biological functions, it will be imperative to continue to develop technologies so that we may better understand the “dark matter” generated by unbiased metabolomics approaches.

**Recommendation 3: determine specific context-dependent responses to biotics.** Biotic interventions can have very different effects in the small bowel versus colon and in the lumen versus at the mucosal surface. They might affect the function of barriers, including the intestinal epithelial and blood-brain barriers, and they often have effects in organs very distant to the intestine including the brain. Biotics can influence both local and systemic immunity through crosstalk that can depend upon developmental and life stages. Context-dependence also demands close attention to the nutrient niche; For instance, the effects of probiotics are greatly influenced by the individual's usual diet, and some probiotics must be administered with a prebiotic component. Other confounding effects, including host genetics and environmental influences, should be carefully considered.

**Recommendation 4: explore a wide range of bioactive microbial metabolites (e.g., not just fermentation of dietary polysaccharides into SCFAs)**. Prebiotic fiber is the most studied class of fermentation substrate for both probiotics and commensal gut bacteria. However, a wide range of substrates can be metabolized into biologically active products. These include microbial fermentation of glutamate into the neurotransmitter GABA, conversion of liver-derived primary bile acids into secondary bile acids, transformation of dietary branched-chain amino acids into branched-chain fatty acids,^155^ and even metabolism of dietary polyphenols and sex hormones. These microbial functions have the potential to impact human health in positive and negative ways.

**Recommendation 5: leverage new approach methods (NAMs) to increase predictive accuracy for human health outcomes**. As technology evolves, using systems that complement traditional transformed cell culture and mouse models will accelerate the path to clinical effectiveness for biotics. Advanced computational techniques including machine learning and artificial intelligence can identify informative patterns among clinical and multi-omic data sets. Human-derived tissues can be interrogated *ex vivo* in perfused organ systems and precision-cut tissue slices, and *in vitro* techniques including organoid models and microphysiological systems can confirm the relevance of preclinical findings to humans. Although these approaches complement clinical trials, in most cases animal models will be needed to investigate inter-organ communication and test hypotheses regarding causality.

## Conclusion

In the past, biotic therapies were selected based on historical and logistical considerations rather than mechanisms of effect. The field is advancing toward a future in which targeted selection of biotics tailored to specific immune, metabolic, neuro-endocrine, commensal microbial, or gut–brain signatures could increase the likelihood of biotics contributing meaningfully to the prevention and treatment of myriad human diseases. Elucidating additional interactions between biotics, diet, and the host will facilitate the rational selection of therapeutic regimens that are more likely to produce health benefits in human trials and, ultimately, in clinical practice.

## Data Availability

No datasets were used or generated.
